# Mapping the contribution of β3-containing GABA_A _receptors to volatile and intravenous general anesthetic actions

**DOI:** 10.1186/1471-2210-7-2

**Published:** 2007-02-24

**Authors:** Anja Zeller, Margarete Arras, Rachel Jurd, Uwe Rudolph

**Affiliations:** 1 Institute of Pharmacology and Toxicology, University of Zürich, Winterthurerstr. 190, CH-8057 Switzerland; 2 Institute of Laboratory Animal Science, University of Zürich, Winterthurerstr. 190, CH-8057 Switzerland; 3 Laboratory of Genetic Neuropharmacology, McLean Hospital, Harvard Medical School, Belmont, MA 02478, USA; 4 Ernest Gallo Clinic and Research Center, University of California, San Francisco, 5858 Horton Street, Suite 200, Emeryville, CA 94608, USA

## Abstract

**Background:**

Agents belonging to diverse chemical classes are used clinically as general anesthetics. The molecular targets mediating their actions are however still only poorly defined. Both chemical diversity and substantial differences in the clinical actions of general anesthetics suggest that general anesthetic agents may have distinct pharmacological targets. It was demonstrated previously that the immobilizing action of etomidate and propofol is completely, and the immobilizing action of isoflurane partly mediated, by β3-containing GABA_A _receptors. This was determined by using the β3(N265M) mice, which carry a point mutation known to decrease the actions of general anesthetics at recombinant GABA_A _receptors. In this communication, we analyzed the contribution of β3-containing GABA_A _receptors to the pharmacological actions of isoflurane, etomidate and propofol by means of β3(N265M) mice.

**Results:**

Isoflurane decreased core body temperature and heart rate to a smaller degree in β3(N265M) mice than in wild type mice, indicating a minor but significant role of β3-containing GABA_A _receptors in these actions. Prolonged time intervals in the ECG and increased heart rate variability were indistinguishable between genotypes, suggesting no involvement of β3-containing GABA_A _receptors. The anterograde amnesic action of propofol was indistinguishable in β3(N265M) and wild type mice, suggesting that it is independent of β3-containing GABA_A _receptors. The increase of heart rate variability and prolongation of ECG intervals by etomidate and propofol were also less pronounced in β3(N265M) mice than in wild type mice, pointing to a limited involvement of β3-containing GABA_A _receptors in these actions. The lack of etomidate- and propofol-induced immobilization in β3(N265M) mice was also observed in congenic 129X1/SvJ and C57BL/6J backgrounds, indicating that this phenotype is stable across different backgrounds.

**Conclusion:**

Our results provide evidence for a defined role of β3-containing GABA_A _receptors in mediating some, but not all, of the actions of general anesthetics, and confirm the multisite model of general anesthetic action. This pharmacological separation of anesthetic endpoints also suggests that subtype-selective substances with an improved side-effect profile may be developed.

## Background

The introduction of general anesthetics into medical practice 160 years ago has revolutionized surgical practice, however, the mechanisms of action of this class of drugs are still only poorly understood. Although general anesthetics have been shown to modulate the activity of a number of proteins, e.g. ligand-gated ion channels [1] and two-pore domain potassium channels [2]*in vitro*, the identification of targets mediating specific actions of general anesthetics *in vivo *has only just begun.

GABA_A _receptors are pentameric ligand-gated ion channels, with the majority of them containing two α, two β and one γ subunit [3]. Mutagenesis studies have identified amino acid residues in GABA_A _receptor β subunits (e.g. N265 in the β3 subunit) to be crucial for the actions of the general anesthetics propofol and etomidate *in vitro *[4-8].

It has been shown that β3(N265M) mice are insensitive to the immobilizing and respiratory depressant action of etomidate and propofol and have a reduced sensitivity for the hypnotic action of these drugs [9,10], suggesting that β3-containing GABA_A _receptors mediate these actions, while etomidate retains its sedative (motor depressant) action at subanesthetic doses. In line with these findings, β2(N265S) mice are still sensitive to the immobilizing and hypnotic actions of etomidate, but lack the sedative response to low doses of etomidate [11].

Inhalation anesthetics like isoflurane show a wider range of targets *in vitro*, including the GABA_A _receptor, glycine receptor, 5-HT3 receptor, kainate receptor, nicotinic acetylcholine receptor, AMPA receptor, and NMDA receptor [1]. It was recently shown that the inhalational anesthetics isoflurane, enflurane and halothane exert their immobilizing action only partly via β3-containing GABA_A _receptors [9,12-14], suggesting that other targets of these volatile anesthetics mediate most of their immobilizing action. The extinction of the conditioned fear response by isoflurane, which is related to the amnestic action of isoflurane, on the other hand, has been suggested to be mediated by cortical α1-containing GABA_A _receptors [15], which are frequently associated with β2 subunits [16].

In this study, we assessed the effects of isoflurane on heart rate, core body temperature and the ECG, the anterograde amnesic action of propofol, and the effects of propofol and etomidate on the ECG. We further assessed the immobilizing and hypnotic action of etomidate and propofol in mice carrying the β3(N265M) mutation on congenic C57BL/6J and 129X1/SvJ backgrounds to confirm the phenotype of the β3(N265M) mutation on two additional genetic backgrounds.

## Results

### The heart rate depressant effect of isoflurane is present but reduced in β3(N265M) mice

**Figure 1:**
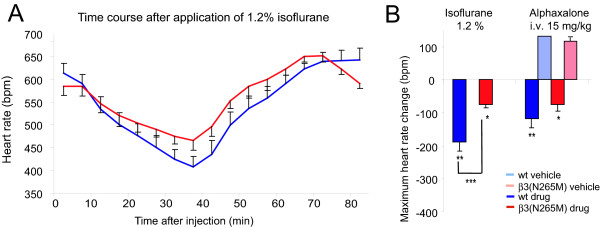
**Isoflurane-induced heart rate depression**. **A**. After application of isoflurane heart rate (HR) decreases in both wild-type and β3(N265M) mice. **B**. Maximum HR change after application of isoflurane compared to 1 hour baseline before application. For comparison, values for alphaxalone, a neurosteroid whose action at the GABA_A _receptor is not influenced by the β3(N265M) point mutation, are displayed as well [10]. Isoflurane: n = 7, alphaxalone i.v.: wt n = 6, β3(N265M) n = 6. * p < 0.05, ** p < 0.01, *** p < 0.001.

It has previously been shown that the immobilizing action of isoflurane is mediated only in part by β3-containing GABA_A _receptors [12,13]. We now investigated whether the heart rate depressant action of isoflurane is dependent on β3-containing GABA_A _receptors. We chose a concentration of 1.2% isoflurane, which represents approximately 0.7 "MAC" in wild type mice and 0.6 "MAC" in β3(N265M) mice with respect to the loss of the hindlimb withdrawal reflex [12]. After application of 1.2% isoflurane for 40 min, the heart rate of wild type mice decreased from a baseline value of 613 ± 18 bpm to 408 ± 27 bpm after 35 min of isoflurane application (-34%, p < 0.001), whereas in β3(N265M) mice it decreased from a baseline value of 585 ± 13 to 466 ± 9 bpm (-21%) (p < 0.01) (Fig. 1). The time course of heart rate decrease is similar in wild type and β3(N265M) mice. The decrease of the heart rate after isoflurane is significantly smaller in β3(N265M) mice compared to wild type mice under these experimental conditions (p < 0.001), however, the difference is rather small and thus the heart rate depressant action of isoflurane is largely mediated by targets other than β3-containing GABA_A _receptors. Alphaxalone, whose action is not affected by the point mutation [7], and which was used as a negative control, displayed no genotype difference with respect to heart rate depression [10], indicating that the heart rate depressant action as such is not affected by the point mutation.

### The hypothermic effect of isoflurane is present but reduced in β3(N265M) mice

**Figure 2:**
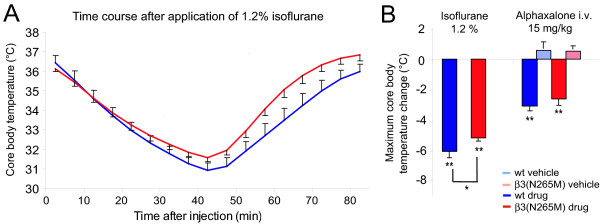
**Isoflurane-induced hypothermia**. **A**. After application of isoflurane core body temperature (CBT) decreases in both wild-type and β3(N265M) mice. **B**. Maximum CBT change after application of isoflurane compared to 1 hour baseline before application. For comparison, values for alphaxalone are displayed as well [10]. Isoflurane: n = 7, alphaxalone i.v.: wt n = 6, β3(N265M) n = 6. * p < 0.05, ** p < 0.01.

Isoflurane decreases the core body temperature (CBT). When mice are placed in a chamber with 1.2% isoflurane (for 40 minutes), the CBT starts to decrease within 5 minutes. The CBT decreased from 36.4 ± 0.4°C and 36.1 ± 0.1°C to 30.9 ± 0.4°C (p < 0.001) and 31.6 ± 0.2°C (p < 0.001) (-16% and -12%) after 40 min of isoflurane application in wild type and mutant mice, respectively (p < 0.05 between genotypes) (Fig. 2). The time course of hypothermia is similar in wild type and β3(N265M) mice. Immediately after the mouse is taken out of the isoflurane chamber, the CBT increases again. The decrease of CBT in the presence of isoflurane is pronounced in both genotypes although slightly but significantly smaller in β3(N265M) mice, indicating a minor role for the β3-containing GABA_A _receptors in this drug action. The hypothermic response to alphaxalone was not different between β3(N265M) and wild type mice, indicating that β3(N265M) mice respond properly to a hypothermic challenge.

### Effects of isoflurane on ECG parameters

**Figure 3:**
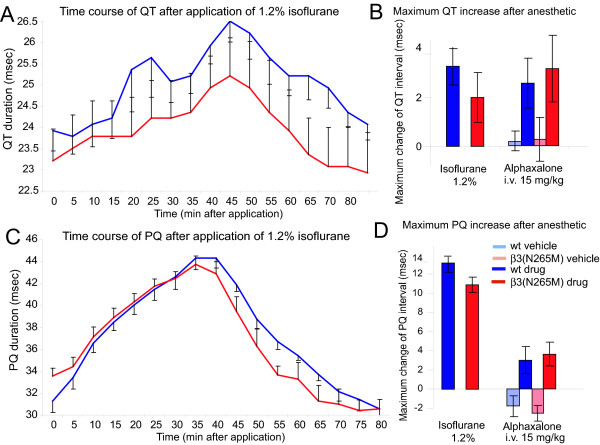
**Isoflurane-induced changes of ECG intervals**. **A, C**. With application of isoflurane, QT and PQ intervals are prolonged. The prolongation is slightly less pronounced in β3(N265M) mice compared to wild type. **B, D**. Maximum change of PQ and QT after application of isoflurane compared to 1 hour baseline before application. For comparison, values for alphaxalone are displayed as well. Isoflurane: n = 7; alphaxalone i.v.: wt n = 6, β3(N265M) n = 6.

General anesthetics are known to alter ECG parameters in humans. In mice, ECG changes have only been recorded for ketamine. We determined the effects of isoflurane on the ECG in wild type and β3(N265M) mice (Fig. 3, Table 1). In wild type mice, isoflurane increased heart rate variability, the PQ, QT, and QRS intervals. There was no genotype difference in these parameters, suggesting no role of β3-containing GABA_A _receptors in the effects of isoflurane on these ECG parameters. There were also no genotype differences after alphaxalone, indicating an unaltered responsiveness of β3(N265M) mice to changes in the ECG.

**Table 1:** Effects of isoflurane-induced anesthesia on baseline ECG parameters.

	Baseline		Isoflurane 1.2%		Alphaxalone 15 mg/kg i.v.	
	wt	β3(N265M)	wt	β3(N265M)	wt	β3(N265M)

RR(msec)	107 ± 3.6	108 ± 4.7	176 ± 129	128 ± 6.3	147 ± 9.7**	132 ± 2.9*
HRV(msec)	5.2 ± 0.8	5.4 ± 0.8	22 ± 2.4**	19 ± 2.7**	18.1 ± 4.1*	12.3 ± 1.6*
QT(msec)	23.5 ± 0.6	19.7 ± 0.9	26.6 ± 0.8*	24.9 ± 1*	26.6 ± 0.8	24.9 ± 1
QRS(msec)	12.2 ± 0.5	11.2 ± 0.5	14 ± 0.8*	13.0 ± 1.2	14 ± 0.8	13 ± 1.2
PQ(msec)	31.8 ± 1.3	32.2 ± 0.7	44.6 ± 1**	43.9 ± 0.7*	44.6 ± 1*	43.9 ± 0.8*

All values are mean ± SEM. RR inter-beat-interval. Group sizes: isoflurane: wt n = 7, β3(N265M) n = 7; alphaxalone i.v.: wt n = 6, β3(N265M) n = 6. * p < 0.05, ** p < 0.01, compared to baseline.

### β3(N265M) mice on different genetic backgrounds show similar response to propofol and etomidate

**Figure 4:**
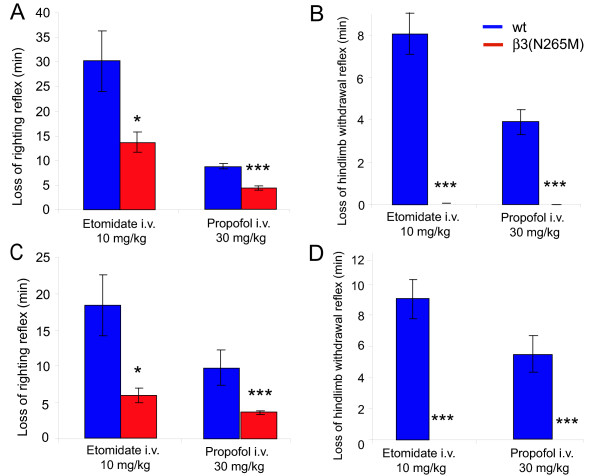
**Behavioural responses to propofol and etomidate in β3(N265M) and wild type mice on pure C57/Bl6J or 129X1/SvJ background**. A, C. Reduction in the duration of the loss of righting reflex (LORR) induced by etomidate and propofol in A) C57/Bl6J β3(N265M) and C) 129X1/SvJ β3(N265M) mice is not influenced by the genetic background. B, D. Failure to induce loss of hindlimb withdrawal reflex induced by propofol and etomidate in B) C57/Bl6J β3(N265M) and D) 129X1/SvJ β3(N265M) mice is not influenced by the genetic background. n = 10. * p < 0.05, *** p < 0.001.

In view of extensive literature on the influence of genetic background in genetically modified mice, we checked whether the response of β3(N265M) mice to general anesthetics is influenced by this. All experiments done in these mice published so far [9,10] and all experiments presented so far in this communication were done with mice harbouring the β3(N265M) point mutation on a mixed background of 129X1/Sv × 129/SvJ. To test whether the genetic background influences the response to etomidate- and propofol-induced loss of righting reflex (LORR) and loss of hindlimb withdrawal reflex (LHWR), these mice were backcrossed 9 and 10 times, respectively, either to C57/BL/6J or 129X1/SvJ wild type mice, to obtain the mutation on a congenic background. These mice were tested with etomidate (10 mg/kg i.v.) and propofol (30 mg/kg i.v.) (Fig. 4). After injection of etomidate LORR was 30 ± 6 min and 18 ± 4 min, in the C57BL/6J wild type and 129X1/SvJ wild type mice, respectively. The duration of LHWR was 7.3 ± 0.9 min and 9.1 ± 1.3 min in C57BL/6J and 129X1/SvJ wild type mice, respectively. Similar to what was previously observed in the mixed background 129/Sv × 129/SvJ β3(N265M) mice [9], also in β3(N265M) mice on the C57BL/6J and 129X1/SvJ backgrounds, 10 mg/kg etomidate lead to a significantly decreased LORR (14 ± 2 min, 6 ± 1 min, respectively, p < 0.05, versus wild type) and to an abolished LHWR (0 min on both backgrounds, p < 0.001 versus the corresponding wild type). Thus, we have observed the same phenotype in three different backgrounds, suggesting that it is robust across different genetic backgrounds.

### Propofol induces anterograde amnesia in β3(N265M) mice

**Figure 5:**
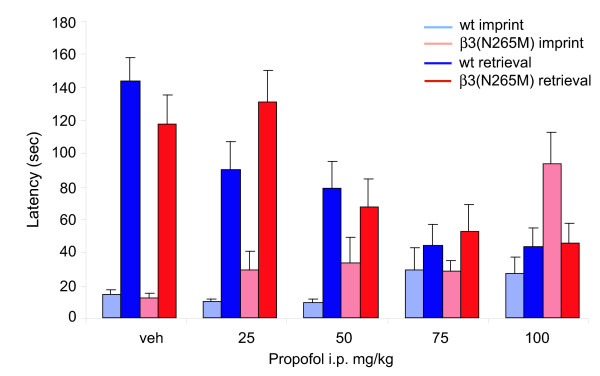
**Propofol induces anterograde amnesia in β3(N265M) mice**. Dose-dependent effect of propofol on imprint and retrieval in both β3(N265M) and wild type mice. n = 15.

General anesthetics are known to induce anterograde amnesia. We tested whether propofol-induced anterograde amnesia is mediated by β3-containing GABA_A _receptors. Increasing doses of propofol (25, 50, 75 and 100 mg/kg i.p.) were employed (Fig. 5). The increasing imprint latency on the training day at the two highest doses of 75 and 100 mg/kg propofol might indicate that in both genotypes mice are slightly sedated at these doses. Most importantly, propofol decreased retrieval, i.e. the latency to re-enter the dark compartment, to a similar degree in wild type and β3(N265M) mice. ANOVA indicated a difference between the drug doses [F(1, 112) = 16.723, p < 0.001 overall, F(4,112) = 4.997, p < 0.001 for drug effect], but no genotype effect [F(1,112) = 3.413, p = 0.067 for genotype effect]. These results indicate that propofol produces anterograde amnesia in both wild-type and β3(N265M) mice and thus by targets independent of β3-containing GABA_A _receptors.

### Effects of etomidate and propofol on ECG parameters

When applying etomidate and propofol via the i.v. route and the i.p. route, we observed a strong decrease in heart rate (see also [10]) and a prolongation of all time domain parameters measured (RR interval, PQ interval, QRS interval, and QT interval (Table 2). The heart rate variability (HRV) increases 2.5 to 8-fold (etomidate 20 mg/kg i.p. and etomidate 10 mg/kg i.v.) in wild type mice and 3 to 5-fold in β3(N265M) mice (propofol 30 mg/kg i.v. and etomidate 10 mg/kg i.v.). The HRV in β3(N265M) mice after injection of etomidate 10 mg/kg i.v. is significantly smaller than HRV in wild type mice after etomidate 10 mg/kg i.v. (p < 0.05). No genotype differences in HRV were noted for all other drug applications. QT, QRS and PQ intervals are prolonged in wild type mice and β3(N265M) mice after injection of etomidate and propofol i.v. and i.p.. There is no genotype effect after injection of any anesthetic for any interval. In summary, as mentioned previously for isoflurane, etomidate and propofol induce changes in ECG parameters, which are largely independent of β3-containing GABA_A _receptors.

**Table 2:** Effects of propofol- and etomidate-induced anesthesia on baseline ECG parameters.

	Baseline		Propofol 30 mg/kg i.v.		Etomidate 10 mg/kg i.v		Propofol 180 mg/kg i.p		Etomidate 20 mg/kg i.p.		Alphaxalone 15 mg/kg i.v.	
	wt	β3(N265M)	wt	β3(N265M)	wt	β3(N265M)	wt	β3(N265M)	wt	β3(N265M)	wt	β3(N265M)

RR(msec)	107 ± 3.6	108 ± 4.7	126 ± 8.5	138 ± 3.2*	205 ± 7***	172 ± 12.8**	283 ± 32**	211 ± 29**	217 ± 7.8	154 ± 12**	138 ± 7.6**	128 ± 6.3*
HRV (msec)	5.2 ± 0.8	5.4 ± 0.8	19.7 ± 3.7	16.8 ± 2.7	40.9 ± 3.3**	27 ± 4.3*#	14.3 ± 0.4*	16 ± 1*	13.2 ± 1.2*	28 ± 5.3*	25.7 ± 6.2*	19 ± 2.7*
QT(msec)	23.5 ± 0.6	19.7 ± 0.9	28.4 ± 1.5*	24.0 ± 1.4	27.4 ± 1.2*	26.0 ± 1.5*	29.8 ± 0.9***	26.8 ± 1.6	27.6 ± 0.9**	32.8 ± 3.7*	26.5 ± 1*	26.5 ± 1.3
QRS (msec)	12.2 ± 0.5	11.2 ± 0.5	13.6 ± 0.8	12.8 ± 1.6	14.6 ± 1*	13.7 ± 1.4	15.3 ± 0.9*	13.2 ± 1	14.2 ± 0.7**	14.6 ± 1.6	13.8 ± 1.1	13.2 ± 1
PQ(msec)	31.8 ± 1.3	32.2 ± 0.7	35.9 ± 2.5	36.4 ± 1.5*	40.4 ± 1.3***	37.4 ± 1.2**	47.8 ± 1.7**	43.8 ± 2**	46.2 ± 2**	44.4 ± 2**	37.3 ± 1.4*	35.8 ± 1.2*

All values are mean ± SEM.. Group sizes: propofol i.v.: wt n = 7, β3(N265M) n = 5, etomidate i.v.: wt n = 7, β3(N265M) n = 7, alphaxalone i.v.: wt n = 6, β3(N265M) n = 6, propofol i.p.: wt n = 4, β3(N265M) n = 6, etomidate i.p.: wt n = 5, β3(N265M) n = 5. * p < 0.05, ** p < 0.01, *** p < 0.001 compared to baseline, # p < 0.05 wild type compared to β3(N265M) mice. Treatment with all different vehicles alone did not lead to statistically significant changes of the ECG parameters recorded compared to baseline. Statistical comparison of drug values with vehicle values and of drug values with baseline values yields similar results.

## Discussion

In this report, we investigated the contribution of β3-containing GABA_A _receptors to various physiological and behavioural endpoints of the inhalational general anesthetic isoflurane and the intravenous general anesthetics propofol and etomidate. We show that the hypothermic and cardiac depressant actions of isoflurane are to a small but significant degree mediated by β3-containing GABA_A _receptors, and that the anterograde amnestic action of propofol is not mediated by β3-containing GABA_A _receptors. We also found that the resistance of the β3(N265M) mice to the immobilizing action of etomidate and propofol and their partial resistance to the hypnotic action of etomidate and propofol are present on a total of three different genetic backgrounds.

We studied the effects of general anesthetics in mice harbouring an asparagine to methionine point mutation in position 265 of the β3 subunit of the GABA_A _receptor. This point mutation renders β3-containing GABA_A _receptors insensitive to the general anesthetics propofol and etomidate, but not to alphaxalone in a recombinant system [7]. The action of the volatile anesthetic enflurane is also strongly reduced by the N265M point mutation in a recombinant system [8,9]. In addition, an *in vivo *increase of the EC_50 _values for enflurane, halothane and isoflurane by 16%, 21%, and 24% has been reported for their immobilizing action in β3(N265M) mice [9,12]. In β3(N265M) mice the suppression of noxious-evoked movements by etomidate and propofol, as measured by the loss of the hindlimb withdrawal reflex was completely abolished and the obtunding or hypnotic response, as determined by the loss of the righting reflex, was also decreased significantly [9]. In addition, the respiratory depressant action of propofol and etomidate was strongly reduced in the β3(N265M) mice [10]. The β3(N265M) mice also display a slightly reduced hypothermia in response to etomidate, but not to propofol [10].

It was previously shown that isoflurane induces LORR and LHWR largely via targets other than β3-containing GABA_A _receptors, although these receptors play an appreciable role in mediating LHWR [12,13]. In this study, we report that core body temperature and heart rate changes in response to isoflurane are also partly mediated by β3-containing GABA_A _receptors, although other targets mediate a much larger part of these responses. The decrease in the heart rate may be secondary to the decrease in body temperature and thus represent a dependent and not an independent parameter. In humans, isoflurane decreases blood pressure by reducing total peripheral resistance while increasing heart rate [17]. Isoflurane depresses both the parasympathetic and sympathetic nervous system, but the hypotension leads to a reflex increase in sympathetic tone, so that isoflurane depresses the parasympathetic nervous system more than the sympathetic nervous system, resulting in tachycardia. Increases in the plasma concentrations of epinephrine and norepinephrine have been found [18], indicating activation of the sympathetic nervous system. Our observation in mice that isoflurane decreases the heart rate might indicate a species difference in the cardiovascular responses to this drug.

We have reported previously that the intravenous general anesthetics etomidate and propofol reduce heart rate in mice [10]. In humans, etomidate and propofol slightly increase the heart rate [19]. In mice, 10 mg/kg etomidate i.v. induces an immediate decrease of both heart rate and core body temperature. The lowest heart rate was observed after 15 minutes, whereas the lowest core body temperature was observed after 30 minutes. At the lowest heart rate, the temperature drop was 4°C, whereas the maximal temperature drop was 5°C [10]. Thus, at a point when the heart rate already starts to recover, the core body temperature is still decreasing.

The time course of heart rate and core body temperature after 1.2% isoflurane presented in this study also shows that the minimal heart rate is reached first, after 35 minutes, and the minimal temperature after 40 minutes. After 35 minutes, the temperature drop was 5.2°C, whereas the maximal temperature drop after 40 minutes was 5.5°C. The heart rate appears to recover before the core body temperature has reached its lowest point, and notably, the heart rate increased before the isoflurane application was stopped, whereas the body temperature only increased after the end of isoflurane application. Thus, for both the intravenous anesthetic etomidate and the volatile anesthetic isoflurane, significant hypothermia develops before the peak drug effects on heart rate are reached, and the time courses of recovery of cardiac depression and hypothermia are different.

Our current and previous studies do not address the question whether heart rate depression and hypothermia are dependent or independent variables, which would require "clamping" of the core body temperature during application of anesthetics. Both the drop in heart rate and the drop in core body temperature both in response to etomidate [10] and isoflurane are largely dependent on targets other than β3-containing GABA_A _receptors and only to a minor degree by β3-containing GABA_A _receptors. The slightly different time course of hypothermia and bradycardia for both etomidate and isoflurane might suggest that these parameters might be at least in part independent. Interestingly, there is no genotype difference between β3(N265M) mice and wild type mice the for drop in heart rate and the drop in core body temperature after alphaxalone/alphadolone [10], which is not affected by the point mutation, indicating that the β3(N265M) display a normal response to an anesthetic challenge. Since mice are small animals, their ratio of body surface to body mass is relatively high and thus hypothermia is expected to be more pronounced than in humans with comparable challenges. It should be noted that the clear species differences especially with respect to changes in heart rate suggest that extrapolation of mouse data to humans should be done with great caution.

General anesthetic agents also alter electrocardiographic (ECG) intervals and decrease heart rate variability in humans as was shown for induction with the barbiturate thiopentone and subsequent inhalation of isoflurane-nitrous oxide [20]. Here, we report that heart rate variability (HRV) is increased by general anesthetics in mice. HRV is considered to be an indicator of cardiac vagal control, and drugs increasing HRV have been shown to reduce mortality and sudden death in severe heart failure in clinical trials [21]. Our finding might indicate that general anesthetics reduces sympathetic tone in mice [22] which results in prolongation of time domain intervals such as QT, QRS and PQ and in an increase in HRV. We showed an increase of HRV after all anesthetics tested, but most pronounced in wild type mice after application of etomidate i.v.. The increase in HRV after etomidate is slightly but significantly reduced in β3(N265M) compared to wild type mice. Prolongation of QT, QRS and PQ intervals is similar in β3(N265M) and wild type mice. To our knowledge, no central mechanisms of HRV regulation have been investigated in mice. In the periphery, β-adrenergic receptors expressed in cardiac tissue are thought to regulate HRV [23]. β1 and β2-ARs play differential roles in the modulation of HRV, each receptor subtype regulating different frequency components of HRV [23]. Etomidate is also known to have agonist activity at α2 adrenergic receptors [24], and it is conceivable that some of its effects on heart rate variability are mediated by these receptors. β3-containing GABA_A _receptors might play a role in the central regulation of HRV. In general, it should be kept in mind that the ECG in the mouse, as performed in this study, is recorded from only two electrodes and information is thus much more limited than information obtained from ECG in humans.

Based on differential sensitivities to propofol of inbred long sleep (ILS) and inbred short sleep (ISS) mice, it has been postulated that a gene responsible for the LORR induced by propofol, termed *Lorp*1, would be located in a 99% confidence interval from 71.4–89.7 Mb on mouse chromosome 7 [25], and in addition, an etomidate-sensitivity quantitative trait locus (QTL) has also been identified in this chromosome region [26,27]. So far, the identity of the *Lorp*1 gene is unknown. Interestingly, the *Gabrb*3 gene encoding the β3 subunit of the GABA_A _receptor is also located on mouse chromosome 7, between 57.4 and 57.7 Mb. To ascertain that the phenotype previously described in 129/Sv × 129/SvJ mice, i.e. partial loss of LORR and complete loss of LHWR in response to etomidate and propofol in β3(N265M) mice [9] is really due to the point mutation in the GABA_A _receptor β3 subunit, the mutant mice were bred for 10 and 9 generations, respectively, onto the 129X1/SvJ and C57BL/6J backgrounds, to yield congenic mice. In all backgrounds examined, we observed the same phenotype, demonstrating that this phenotype is very robust across different backgrounds, and thus that the observed phenotype is really associated with the N265M point mutation in the *Gabrb*3 gene. Thus, our analysis shows that *Gabrb*3 and *Lorp*1 are separate genes.

We further tested β3(N265M) mice on the congenic 129X1/SvJ background in the passive avoidance paradigm, to examine whether the anterograde amnesic action of propofol would be mediated by β3-containing GABA_A _receptors. Our results suggest that this anesthetic endpoint is independent of β3-containing GABA_A _receptors. This result is consistent with previous findings that both the anterograde amnesic action of diazepam [28] (studied using the same paradigm) as well as the anterograde amnesic action of isoflurane (determined as an extinction of conditioned fear response) [15] are mediated by α1-containing GABA_A _receptors. Since α1β2γ2 is the most abundant GABA_A _receptor subtype [29], it is tempting to speculate, and consistent with all data currently available, that this receptor subtype mediates the anterograde amnesic actions not only of sedative-hypnotic agents like diazepam (Rudolph et al., 1999), but also of general anesthetic agents. However, in α5^-/- ^mice, long term potentiation (LTP) in CA1 is reduced by etomidate in wild type but not in α5^-/- ^mice [30]. Furthermore, learning in the Morris water maze and in fear conditioning is impaired by etomidate in wild type mice, but not in α5^-/- ^mice [30]. These data indicate a role for α5-containing GABA_A _receptors in drug-induced amnesia, in addition to its involvement in certain hippocampus-dependent forms of associative learning like trace fear conditioning [30,31].

Our current knowledge on the role of β3-containing GABA_A _receptors in the action of the general anesthetics etomidate, propofol and isoflurane is summarized in Table 3. The genetic dissection of the pharmacological spectrum of general anesthetics is of interest for the design of novel general anesthetic compounds, in which the various desired and undesired effects can be separated.

**Table 3:** Proposed roles of β3-containing GABA_A _receptors in the actions of the general anesthetics etomidate, propofol and isoflurane

	Etomidate	Propofol	Isoflurane
Immobility	+++^9^	+++^9^	+^12^
Hypnosis	++^9,11^	++^9^	+^12^
Respiratory depression	+++^10^	+++^10^	n.d.
Sedation	---^11^	n.d.	n.d.
Anterograde amnesia	n.d.	**---**	n.d.
Hypothermia	+^10^	---^10^	**+**
Heart rate depression	+^10^	---^10^	**+**
ECG changes	**---**	**---**	**---**

+++ designates complete mediation of this action by β3-containing GABA_A _receptors. ++ designates partial mediation (approximately 50%), + designates a small but significant contribution of β3-containing GABA_A _receptors. --- designates no mediation at all or a very minor contribution through β3-containing GABA_A _receptors. n.d. not determined. Data which were taken from the literature are marked with superscripts, which refer to this paper's reference list. Heart rate depression might be secondary to hypothermia.

## Conclusion

We show that β3-containing GABA_A _receptors mediate a small, but significant, part of isoflurane-induced heart rate depression and hypothermia, which is in line with the small but significant contribution of β3-containing GABA_A _receptors to isoflurane-induced immobility and hypnosis reported previously. We also found that isoflurane-induced ECG changes are not mediated by β3-containing GABA_A _receptors. These data indicate that isoflurane exerts its effects via many targets, β3-containing GABA_A _receptors being one of them. Furthermore, we found a dissociation between the immobilizing and anterograde amnestic action of propofol. Whereas as previously shown the immobilizing action of etomidate is mediated by β3-containing GABA_A _receptors, the anterograde amnesic action is independent of this receptor subtype. Prolongation of ECG intervals induced by etomidate and propofol was not mediated by β3-containing GABA_A _receptors. By demonstrating that etomidate- and propofol-induced immobilization is mediated exclusively and hypnosis is mediated partly by β3-containing GABA_A _receptors in two congenic backgrounds, we show that this phenotype is robust across three backgrounds and that the *Gabrb*3 locus is different from the *Lorp*1 locus.

## Methods

### Animals

Generation, characterization and breeding of β3(N265M) mice has been described previously [9]. Mice used for telemetry were 3 months old at the time of surgery, 4 months old at the beginning of the experiments and 10 months at the end of the telemetry experiments. Mice used in the passive avoidance paradigm were 6 to 8 weeks old, and mice used for reflex tests were 4 to 7 months old. Telemetry experiments were performed on 129/Sv × 129X1/SvJ (12.5%/87.5%) mice, anterograde amnesia was performed on a congenic 129X1/SvJ background (10 backcrosses with 129X1/SvJ), and loss of righting reflex and loss of hindlimb withdrawal reflex were performed on a congenic 129X1/SvJ background (10 backcrosses with 129X1/SvJ) and a congenic C57BL/6J background (9 generations of backcrosses with C57BL/6J mice). All animal experiments have been approved by the cantonal veterinary office in Zurich.

### Surgery

16 female mice (8 β3(N265M) mice and 8 wild type controls) were implanted under isoflurane anaesthesia (3–5% in oxygen) with intraperitoneal radiotelemetry transmitters for measuring core body temperature, ECG and activity (model No. ETA-F20, Data Sciences International (DSI), St. Paul, MN). The transmitter body was implanted under sterile conditions in the abdominal cavity and the sensing leads were positioned as described previously [32]. Mice received postoperative antibiotics (20 mg/kg sulfadoxin, 5 mg/kg trimethoprim, Borgal 7.5%, Hoechst Roussel vet, Provet AG, Lyssach, Switzerland) and postoperative pain treatment for 5 days (2.5 mg/kg flunixin s.c., Finadyne, BERNA Veterinärprodukte AG, Berne, Switzerland). Mice were allowed to recover for 4 weeks before the first experiment. To ascertain full recovery after surgery we measured core body temperature and heart rate over 72 h.

### Experimental Conditions

Mice implanted with telemetry transmitters were singly housed in standard laboratory conditions with a 12 h light/dark schedule (lights on 8:00 am, lights off 8:00 pm) and free access to food and water. Experiments were performed between 9 am and 12 am. Mice used for passive avoidance and reflex tests were group housed. The passive avoidance experiment was performed between 9 am and 12 am, the reflex tests between 8 am and 5 pm.

### Effect of Anesthetics on Core Body Temperature (CBT), Heart Rate (HR) and ECG Parameters (PQ, QRS, QT, heart rate variability(HRV))

For drug and vehicle administration experiments, a baseline was recorded between 0 and 2 hours after lights on and drugs were administered immediately afterwards. Drug effects were compared to vehicle effects, which did not differ significantly from baseline, except for isoflurane, where the drug effect was compared to baseline. Mice were treated (in this order) with 30 mg/kg propofol i.v. (Sigma-Aldrich Chemicals, Buchs, Switzerland), 10 mg/kg etomidate i.v. (Janssen-Cilag, Neuss, Germany), 15 mg/kg alphaxalone i.v. (Saffan^®^, alphaxalone/alphadolone 15/5 mg/kg), isoflurane 1.2% in air (Arovet, Zollikon, Switzerland), pentobarbital 60 mg/kg i.p. (Nembutal, Abbott AG, Baar, Switzerland/Abbott Laboratories, Chicago, USA), 180 mg/kg propofol i.p., 20 mg/kg etomidate i.p. (see also [10]). Vehicle solutions were as follows: propofol, 14% Cremophor EL, etomidate 35% propylene glycol, Saffan^® ^0.9% saline. The doses used for the i.v. route have been previously examined for their effects on loss of reflexes [9]. Intravenous injections were performed in the tail vein after warming the tail in 39°C warm water to achieve vasodilatation. The doses for the i.p. route were determined in pre-tests. The conditions for isoflurane application were chosen to ensure that animals would not die of hypothermia, since animals were not warmed up with an external heating source. For assessment of the effect of isoflurane, a sealed Plexiglas chamber was used as described previously [12]. Only one mouse at one time was placed into the chamber to avoid overlapping of the radio transmitter signals. After turning on the transmitters with a magnet, a one hour baseline was measured with data sampling for 30 s every 3 minutes. Five minutes before injection the sampling schedule was switched to continuous ECG recording and body temperature and heart rate were sampled every 30 s. Two hours after the return of the righting reflex the continuous sampling was switched to a data sampling for 30 s every 3 minutes and then continued for another 15 hours. Data were acquired with the Dataquest ART 3.0 acquisition system (DataSciences International, St. Paul, MN, USA). All signals (CBT, HR and ECG parameters) were recorded simultaneously in the same experiment. CBT and HR were calculated by the acquisition software (Dataquest A.R.T. 3.01, Data Sciences International). The ECG signal was further processed to derive time domain parameters (PQ, QRS, QT) with the Physiostat™ ECG Analysis 4.00 (DataSciences International) software.

### Amnestic effect of propofol in a passive avoidance task

The amnestic effect of propofol was tested in a single-trial passive avoidance task. Propofol was injected 20 min before training at 0, 25, 50, 75 and 100 mg/kg i.p. After injection, mice were put back in their home cage and after 20 min, when the drug effect was fully developed, they were placed in a lit chamber (860 lux light intensity). Mice were allowed to explore the chamber for 30 sec, then a door was opened to a dark chamber and latency of the mouse to enter the dark chamber was measured (imprint). The door was closed and 10 sec later two foot shocks with 0.5 mA strength at an interval of 3 sec were applied. Retention was tested 24 h after training as latency to re-enter the dark chamber (retrieval). The experimenter was blinded to genotype and substance.

### Statistical Analysis

Results are expressed as mean ± SEM. For analysis of telemetry data statistical differences were assessed by using the paired Student's t-test for testing whether the effect of anesthetic is significant compared to the vehicle, and the unpaired Student's t-test for determining potential genotype differences between wild type and mutant mice. The minimum CBT or HR after injection of anesthetic and the mean of vehicle values over a time period of two hours after injection were determined and compared to the mean of one hour baseline before injection. For analysis of passive avoidance data, repeated measures ANOVA followed by a post hoc Bonferroni test was used. For analysis of reflex data, the unpaired Student's t-test was used.

## List of abbreviations

GABA γ-aminobutyric acid

GABA_A _receptor GABA type A receptor

HR heart rate

CBT core body temperature

ECG electrocardiogram

LTP long term potentiation

## Authors' contributions

AZ designed and carried out telemetry experiments, reflex tests, passive avoidance test, analysed all data and drafted the manuscript.

MA consulted on telemetry experiments and statistical analysis.

RJ generated the β3(N265M) mouse model and participated in the preparation of the manuscript.

UR conceived the study, and participated in its design and coordination and helped to draft the manuscript. All authors read and approved the final manuscript.
